# Inter-State Medical Certificates

**Published:** 1896-06

**Authors:** 


### INTER-STATE MEDICAL CERTIFICATES.

The following editorial in the Philadelphia Polyclinic has force respecting dental certificates. It is published in full.

R. G.

Since the establishment of State Examining and Licensing Boards in the United States, an anomaly, both amusing and vexatious, has been exhibited. American physicians who feel aggrieved at the fact that they could not practise their profession in foreigh countries without previous examination, are now debarred from "all the rights and privileges" their diplomas are supposed to confer upon them if they but cross a river, or an imaginary boundary line in their own country. A physician living in Philadelphia, though he be of eminence and acknowledged repute in this country and Europe, if he had neglected to register in the state of New Jersey previous to a certain date fixed by its Medical Practice Law, could not lawfully prescribe for one of his own patients temporarily sojourning at Atlantic City, unless summoned in consultation by a physician qualified under the laws of New Jersey. Physicians who may desire to remove from Pennsylvania to New York or *vice versa* must undergo the nuisance of examinations; and these examinations while perhaps well suited to test the knowledge of recent graduates are hardly of the proper character for physicians who have not recently attended lectures but are nevertheless thoroughly competent at the bedside.

This state of affairs is radically wrong and should not be permitted to continue. The various licensing authorities should agree upon the conditions necessary to permit the certificates of one board to be accepted by all other boards, while physicians possessing the diplomas of reputable schools issued before the days of State Examinations in any State upon proper authentication by the licensing authority. The subject is one of considerable importance, and the interests of more than a few individuals are involved. We should be glad to see it thoroughly discussed and a practical conclusion reached and put into effect, which shall properly safeguard the public without requiring physicians either to pass their lives in the State they may happen to begin practice in, or to undergo forty-odd examinations when jnst fresh from college.

